# A liver function test pathway significantly increases the early detection of chronic liver disease and cirrhosis

**DOI:** 10.1097/HC9.0000000000000887

**Published:** 2026-01-29

**Authors:** Jingwei Gao, Haroon Ahmed, Rebecca Cannings-John, Ashley Akbari, Aled Davies, Thomas Peter I. Pembroke

**Affiliations:** 1 Population Data Science, Swansea University Medical School, Faculty of Medicine, Health & Life Science, Swansea University, Swansea, UK; 2 Division of Population Medicine, Cardiff University, Neuadd Meirionnydd, Heath Park, Cardiff, UK; 3 Centre for Trials Research, Cardiff University, Neuadd Meirionnydd, Heath Park, Cardiff, UK; 4 PRIME Centre Wales, Cardiff University, Neuadd Meirionnydd, Heath Park, Cardiff, UK; 5 Department of Gastroenterology and Hepatology, University Hospital of Wales, Cardiff, UK

**Keywords:** aspartate aminotransferases, clinical pathways, liver cirrhosis, liver diseases, primary health care

## Abstract

**Background:**

To enhance early liver disease detection, a clinical pathway integrating reflex AST testing and automated AAR reporting was implemented. We aim to evaluate the long-term effectiveness of introducing reflex AST testing by assessing its impact after implementation in 2 regions of Wales.

**Methods:**

We applied a quasi-experimental, Difference-in-Difference approach to evaluate the introduction of the reflex AST:ALT pathway in Wales (January 2010 to December 2023). Outcomes were the monthly incidence rate of (1) chronic liver disease (including cirrhosis) and (2) cirrhosis in the 2 intervention regions versus the control regions.

**Results:**

In total, 78,917 individuals with liver disease were included in the study. A significant increase in cirrhosis diagnoses was observed in both regions (first region: incidence rate ratio=1.24, 95% CI: 1.15–1.34, *p*<0.001; second region: incidence rate ratio=1.16, 95% CI: 1.02–1.33, *p*=0.028). The incidence of composite chronic liver disease (including cirrhosis) increased transiently in the second region only (incidence rate ratio=1.35, 95% CI: 1.16–1.56, *p*<0.001).

**Conclusions:**

In this long-term, population-level evaluation, reflex AST:ALT testing increased cirrhosis detection in both regions and produced a short-term rise in chronic liver disease (including cirrhosis) diagnoses one region, strengthening the evidence of the pathway’s effect on cirrhosis detection. Further study is warranted to understand regional variation.

## Graphical abstract

**Figure:**
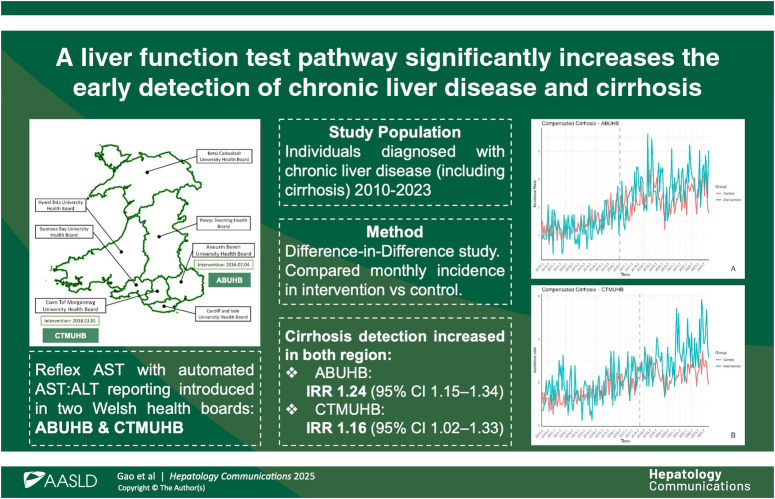

## INTRODUCTION

Chronic liver disease (CLD) poses a significant global health and economic burden, accounting for approximately 2 million deaths annually.^1^^,^^2^ In the United Kingdom, liver disease mortality has continued to rise over the past three decades, in contrast to other major diseases where mortality has declined.^3^ It has become the third leading cause of premature death and one of the most common causes of mortality in working-age individuals.^4^^–^^6^

CLD progresses through the stages of fibrosis, with severity significantly associated with liver-related outcomes. Once cirrhosis develops, the burden on health care systems rises sharply due to increased complications and costs.^7^^–^^9^ Early detection of liver disease is essential for timely intervention to prevent progression and reduce disease burden.^3^^,^^10^ Key time points in the early detection of liver disease are (1) prior to the development of cirrhosis, and (2) prior to hepatic decompensation if cirrhosis is established. There is a growing focus on noninvasive fibrosis assessment tools, such as transient elastography and serum biomarkers, which have been widely validated and are well accepted in clinical practice.^11^ Among these, indirect biomarkers, which are readily measurable biochemical parameters in peripheral blood, offer a cost-effective and easily accessible alternative during routine clinical visits. Consequently, indirect biomarkers have been extensively developed and applied to identify individuals with cirrhosis without the need for invasive liver biopsy. One of the most established and widely available markers of hepatocyte stress and apoptosis is the AST/ALT ratio (AAR), which has shown a positive predictive value of 93% when using AAR>1 to detect cirrhosis in patients with HCV and NAFLD.^12^^,^^13^

Over the past decade, several community-based pathways have been introduced to improve risk stratification and early liver disease diagnosis.^14^ One such initiative is the introduction of reflex AST testing to enable AAR reporting and prompt referral through the Gwent AST project in 2 regions of Wales in 2016 and 2018. This approach allows the reflex use of AST testing for abnormal ALT results, enabling automatic AAR reporting and prompt onward referral for further assessment.^15^ Following the implementation of the reflex AST testing, we demonstrated an 81% increase in the new diagnosis of cirrhosis within 2 years in Aneurin Beven University Health Board (ABUHB), one of the first Wales regions to adopt this approach.^15^ However, our previous work study lacked a control arm, making it difficult to determine whether the observed increase was directly attributable to the intervention or influenced by other factors, such as underlying trends in liver disease incidence. In addition, the long-term sustainability and broader impact of these pathways remain uncertain. Furthermore, with the same approach later introduced in more Welsh regions, it is also unclear whether the intervention would produce a consistent effect across different geographical settings.

The objective of this study is to assess the long-term effectiveness of the introduction of reflex AST testing by evaluating its impact in 2 Welsh regions where the intervention was implemented: ABUHB and Cwm Taf Morgannwg University Health Board (CTMUHB). We aimed to quantify the changes in the detection of CLD (including compensated cirrhosis) and cirrhosis, providing valuable insights into the sustained impact of community-based liver disease detection pathways on the early diagnosis of liver disease.

## METHODS

### Setting and data source

We used the Secure Anonymised Information Linkage (SAIL) Databank, which contains anonymized, individual-level routinely collected linked electronic health record data for the Welsh population.^16^^–^^19^ The SAIL Databank includes secondary care data for the whole Welsh population and primary care data for approximately 86% of the Welsh population.^20^ To provide a comprehensive assessment of liver disease cases, we combined data from primary and secondary care records. Primary care data were accessed from the Welsh Longitudinal General Practice data, which collects event histories for people registered with a SAIL-supplying general practice in Wales and employs the Read version 2 clinical coding system. Secondary care data, including inpatient admissions (emergency, elective, and maternity) and day-care procedures, were retrieved from the Patient Episode Database for Wales and coded according to the International Classification of Diseases, 10th Revision (ICD-10). In addition, we obtained demographic and deprivation data from the Welsh Demographic Service Dataset, using the 2019 Welsh Index of Multiple Deprivation quintiles to assess area-level deprivation based on residential locations within the 2011 Lower-layer Super Output Area boundaries. Individual health board allocations were determined based on the health board information available in the Welsh Longitudinal General Practice and Patient Episode Database for Wales data sources.

### Population

All individuals with available residency information and registration with a SAIL-contributing general practice were included in the study using a unique anonymized individual identifier known as an Anonymised Linkage Field.^16^^,^^17^ The study population comprised individuals identified from primary and secondary care settings and diagnosed with CLD (including cirrhosis) across the 7 health boards in Wales—ABUHB, CTMUHB, Betsi Cadwaladr University Health Board (BCUHB), Cardiff and Vale University Health Board (CVUHB), Hywel Dda University Health Board (HDUHB), Powys Teaching Health Board (PTHB), and Swansea Bay University Health Board (SBUHB)—between January 1, 2010, and December 31, 2023. CLD (including cirrhosis) was defined as a combination of diagnoses comprising both (1) noncirrhotic chronic liver conditions (chronic etiologic conditions and hepatic fibrosis without cirrhosis) and (2) compensated cirrhosis (including portal hypertension prior to decompensation), as per our previous work.^21^^,^^22^ Chronic etiologic conditions include alcohol-associated liver disease, NAFLD, metabolic liver disease, HBV, HCV, autoimmune liver disease, hemochromatosis, unspecified hepatitis, congestive hepatopathy, toxic liver disease, and miscellaneous conditions. Individuals diagnosed with decompensation (including chronic hepatic failure, unspecified hepatic failure, hepatorenal syndrome, esophageal varices with bleeding) or HCC were excluded, as this pathway aims to detect individuals with cirrhosis before decompensation. A list of ICD-10 and Read v2 codes to identify individuals with liver disease is provided in Supplemental Table S1, http://links.lww.com/HC9/C226.

### Intervention and control

**FIGURE 1:**
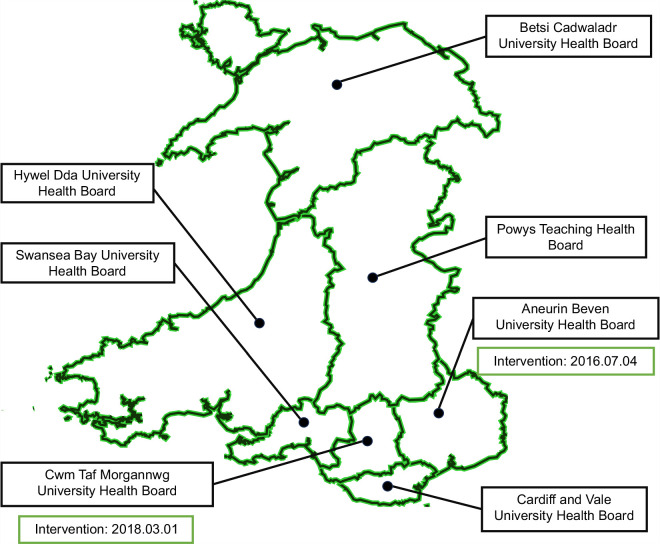
Map of the seven health boards in Wales. This map illustrates the geographical boundaries of the seven regional health boards responsible for health care delivery in Wales: Aneurin Bevan University Health Board (ABUHB), Cwm Taf Morgannwg University Health Board (CTMUHB), Betsi Cadwaladr University Health Board (BCUHB), Cardiff and Vale University Health Board (CVUHB), Hywel Dda University Health Board (HDUHB), Powys Teaching Health Board (PTHB), and Swansea Bay University Health Board (SBUHB). ABUHB, covering the Gwent area in southeast Wales—including Newport, Caerphilly, and Abergavenny—was the first to implement the Gwent AST Project on July 4, 2016, positioning it as a leader in liver disease detection innovation. CTMUHB, serving Rhondda Cynon Taf, Merthyr Tydfil, and Bridgend, later adopted the Gwent AST Project in March 2018.

The introduction of the reflex AST testing and AST:ALT calculation is a diagnostic strategy designed to improve the early detection of significant liver disease. Implemented through the Gwent AST project under the Wales Liver Plan, the pathway automatically identifies patients who may require further assessment. When a patient presents with an elevated ALT level, a reflex AST test will be performed, with the AAR calculated. If the ratio is ≥1, the general practitioners will be advised to refer the patient for fibrosis assessment—typically using transient elastography (FibroScan).^16^ This provides an opportunity to make a liver disease diagnosis without cirrhosis and a window for lifestyle advice intervention in primary care without the need for onward referral to secondary care. The promotion of reflex AST testing was initially introduced in ABUHB, a local health board in Wales, on July 4, 2016, and then implemented in CTMUHB, a neighboring local health board, in March 2018 (Figure 1). Therefore, we consider ABUHB and CTMUHB as intervention groups, and the remaining 5 health boards (BCUHB, CVUHB, HDUHB, PTHB, and SBUHB) as control groups for the time period under investigation in this study. The preimplementation period was defined as January 1, 2010, to July 3, 2016, for ABUHB, and January 1, 2010, to February 28, 2018, for CTMUHB. The postimplementation periods were defined as July 4, 2016, to December 31, 2023, for ABUHB and March 1, 2018, to December 31, 2023, for CTMUHB.

### Outcomes

Although the reflex AST/AST:ALT pathway was introduced to facilitate identification of cirrhosis before decompensation, in clinical practice, an elevated AAR may trigger further evaluation, and many patients are often first coded with etiologic conditions instead of cirrhosis. Therefore, we speculate that this pathway may promote consideration of the underlying cause of liver disease, and defined a boarder primary composite outcome as CLD (including cirrhosis), consisting of noncirrhotic and cirrhosis liver disease in our study. Accordingly, our primary outcome was defined as the monthly incidence rate of CLD (including cirrhosis) recorded in primary or secondary care. To evaluate the pathway’s intended target more specifically, we conducted a prespecified subanalysis of the monthly incidence of compensated cirrhosis alone. The monthly incidence rate was calculated for each health board by dividing the number of new cases by the total population of the corresponding health board, and reported per 100,000 inhabitants. A new case was defined as an individual receiving a first diagnosis during the study period (January 1, 2010, to December 31, 2023) with no prior liver disease history from January 1, 1994, to December 31, 2009. To capture the most clinically relevant etiology, only the most advanced stage of diagnosis on the date of the incident event was considered. Population data for Wales were obtained from the Office for National Statistics (ONS) population estimates.^23^

### Statistical analysis

Descriptive analyses were conducted to summarize the demographic and socioeconomic characteristics of the individuals diagnosed with CLD (including cirrhosis). The average incidence rates and corresponding SD were calculated in the intervention and control groups during the preimplementation and postimplementation periods of reflex AST testing.

A Difference-in-Difference (DiD) analysis was employed to test the impact of the intervention on the early detection of liver disease by determining whether there was a statistically significant difference in the change in each outcome. The analysis compared changes of incidence rates of CLD (including cirrrhosis) and cirrhosis from preimplementation to postimplementation for the intervention group relative to the control groups. The Poisson regression and linear regression models were fitted to the monthly counts with the corresponding health board population as the offset, with predictors including time, an intervention group indicator, and a postimplementation period indicator. The incidence rate ratio (IRR) and Average Treatment Effect on the Treated (ATT) were constructed to estimate the DiD effect, representing the impact of the intervention on the incidence rate of liver disease. The monthly incidence rates in the intervention and control groups were visualized graphically to detect trends and ensure the existence of preintervention parallel trends, an assumption of quasi-experimental DiD analysis. We tested for parallel trends using the Wald test to assess whether the linear trends were parallel prior to the intervention. Where the preintervention parallel trends assumption was not supported by statistical and visual assessment, the results are interpreted as exploratory, as the violations of this assumption undermine the validity of causal inference in DiD analysis. All analyses were conducted using Stata/SE version 19.0 (StataCorp LLC) and R version 4.3.3 (R Foundation for Statistical Computing) via RStudio version 2025.05.1 (Posit Software).

### Sensitivity analysis

We conducted a sensitivity DiD analysis restricted to data from primary care settings (Welsh Longitudinal General Practice data source) with the same outcomes, to evaluate potential changes in liver disease incidence rates within the primary care context.

### Ethics approval

This study was conducted in accordance with the Declarations of Helsinki and Istanbul, using routinely collected anonymized data available in the SAIL Databank. Access to SAIL data is subject to approval by the independent Information Governance Review Panel, which ensures appropriate and ethical use of the data. This study was approved by the Information Governance Review Pane (Project ID: 1492). All data access was conducted through SAIL’s privacy-protecting Trusted Research Environment. As this study used anonymised secondary data with no direct participant contact, individual informed consent was not required and was waived by the approving review body. The research complied with the ethical guidelines and adhered to the Data Protection Act 2018 to safeguard the privacy and confidentiality of all individuals.

## RESULTS

**FIGURE 2:**
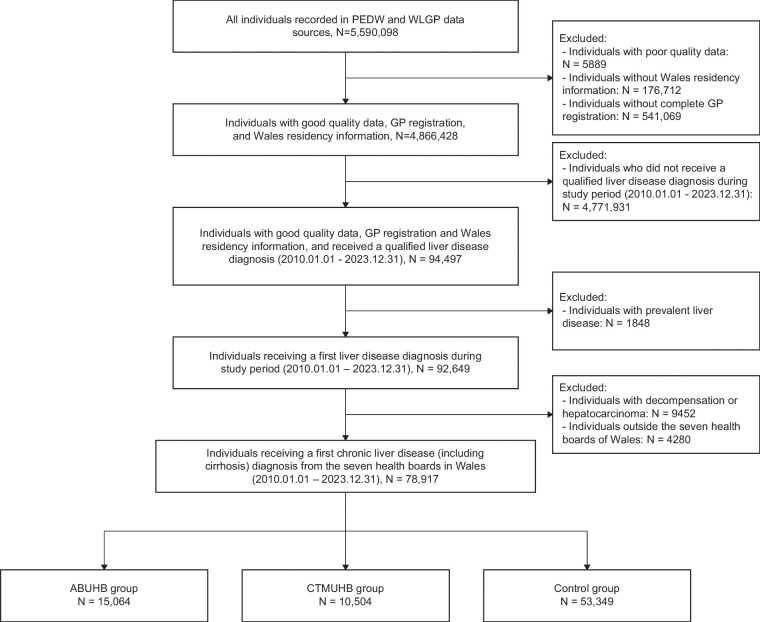
Consort diagram of cohort extraction. This diagram outlines the flow of data sources and participant selection used to construct the final study cohort. Abbreviations: ABUHB, Aneurin Bevan University Health Board; CTMUHB, Cwm Taf Morgannwg University Health Board; GP, general practice; PEDW, Patient Episode Database for Wales; WLGP, Welsh Longitudinal General Practice.

A total of 78,917 individuals with CLD (including cirrhosis) from the seven health boards of Wales between January 1, 2010, and December 31, 2023, were included in the study. Among them, 15,064 (19.09%) and 10,504 (13.31%) incident cases were identified in the ABUHB and CTMUHB groups, respectively, and 53,349 (67.60%) in the control group (Figure 2). The age and sex distributions were similar across groups, with median ages of 58, 57, and 59 years (ABUHB, CTMUHB and control, respectively), and the proportion of males was 48.71%, 52.20%, and 50.48%, respectively. However, a higher percentage of individuals from the most deprived areas was observed in ABUHB and CTMUHB (31.69% and 31.68%, respectively) compared to the control group (20.72%). Demographic and socioeconomic characteristics remained consistent before and after the intervention within each group (Table 1).

**TABLE 1:** Demographic characteristics of intervention and control regions in Aneurin Bevan and Cwm Taf Morgannwg University Health Boards

	ABUHB				CTMUHB			
Demographic characteristics	ABUHB, preimplementation^a^ N=4921	ABUHB, postimplementation^b^ N=10,143	Control regions, preimplementation N=18,226	Control regions, postimplementation N=35,123	CTMUHB, preimplementation N=4159	CTMUHB, postimplementation N=6345	Control regions, preimplementation N=24,835	Control regions, postimplementation N=28,514
Sex								
Male	2453 (49.85)	4884 (48.15)	9176 (50.35)	17,753 (50.55)	2238 (53.81)	3245 (51.14)	12,595 (50.71)	14,334 (50.27)
Female	2468 (50.15)	5259 (51.85)	9050 (49.65)	17,370 (49.45)	1921 (46.19)	3100 (48.86)	12,240 (49.29)	14,180 (49.73)
Age, median (IQR)	58 (25)	59 (25)	59 (25)	60 (24)	57 (25)	59 (24)	59 (24)	60 (24)
Age group (y)								
0–17	44 (0.89)	92 (0.91)	184 (1.01)	376 (1.07)	39 (0.94)	62 (0.98)	246 (0.99)	314 (1.10)
18–29	305 (6.20)	512 (5.05)	1036 (5.68)	1547 (4.40)	252 (6.06)	311 (4.90)	1366 (5.50)	1217 (4.27)
30–39	470 (9.55)	1015 (10.01)	1692 (9.28)	3106 (8.84)	406 (9.76)	661 (10.42)	2242 (9.03)	2556 (8.96)
40–49	797 (16.20)	1279 (12.61)	2621 (14.38)	4655 (13.25)	660 (15.87)	873 (13.76)	3551 (14.30)	3725 (13.06)
50–59	1000 (20.32)	2190 (21.59)	3739 (20.51)	7589 (21.61)	878 (21.11)	1394 (21.97)	5181 (20.86)	6147 (21.56)
60–69	1015 (20.63)	2038 (20.09)	3929 (21.56)	7518 (21.40)	879 (21.13)	1280 (20.17)	5320 (21.42)	6127 (21.49)
70–79	766 (15.57)	1800 (17.75)	2842 (15.59)	6356 (18.10)	682 (16.40)	1110 (17.49)	3995 (16.09)	5203 (18.25)
80+	524 (10.65)	1217 (12.00)	2183 (11.98)	3976 (11.32)	363 (8.73)	654 (10.31)	2934 (11.81)	3225 (11.31)
WIMD 2019 quintiles								
1, most deprived	1549 (31.48)	3225 (31.80)	3952 (21.68)	7104 (20.23)	1395 (33.54)	1933 (30.46)	5398 (21.74)	5658 (19.84)
2	1117 (22.70)	2282 (22.50)	3491 (19.15)	6745 (19.20)	1468 (35.30)	2207 (34.78)	4753 (19.14)	5483 (19.23)
3	1027 (20.87)	1989 (19.61)	3667 (20.12)	7057 (20.09)	567 (13.63)	816 (12.86)	4902 (19.74)	5822 (20.42)
4	669 (13.59)	1437 (14.17)	3638 (19.96)	7183 (20.45)	292 (7.02)	582 (9.17)	4978 (20.04)	5843 (20.49)
5, least deprived	559 (11.36)	1210 (11.93)	3478 (19.08)	7034 (20.03)	437 (10.51)	807 (12.72)	4804 (19.34)	5708 (20.02)
Liver disease stage/etiology^c^								
Chronic liver disease (without cirrhosis)	4311 (87.60)	8699 (85.76)	15,936 (87.44)	30,714 (87.45)	3396 (81.65)	5348 (84.29)	21,696 (87.36)	24,954 (87.51)
Cirrhosis	610 (12.40)	1444 (14.24)	2290 (12.56)	4409 (12.55)	763 (18.35)	997 (15.71)	3139 (12.64)	3560 (12.49)

*Note:* Data are presented as n (%) unless otherwise stated.

^a^ For ABUHB: preimplementation=January 1, 2010, to July 3, 2016; postimplementation=July 4, 2016, to December 31, 2023.

^b^ For CTMUHB: preimplementation=January 1, 2010, to February 28, 2018; postimplementation=March 1, 2018, to December 31, 2023.

^c^ Liver disease stage/etiology: The classification of liver disease stages/etiology is based on our previous work. Chronic liver disease (without cirrhosis) includes alcohol-associated liver disease, NAFLD, metabolic liver disease, hepatitis B, hepatitis C, autoimmune liver disease, hemochromatosis, hepatitis not specified, congestive hepatopathy, toxic liver disease, and miscellaneous hepatic fibrosis; cirrhosis includes cirrhosis and cirrhosis with portal hypertension.

Abbreviations: ABUHB, Aneurin Bevan University Health Board; CTMUHB, Cwm Taf Morgannwg University Health Board; IQR, interquartile range; WIMD, Welsh index of multiple deprivation.

### CLD (including cirrhosis)

**FIGURE 3:**
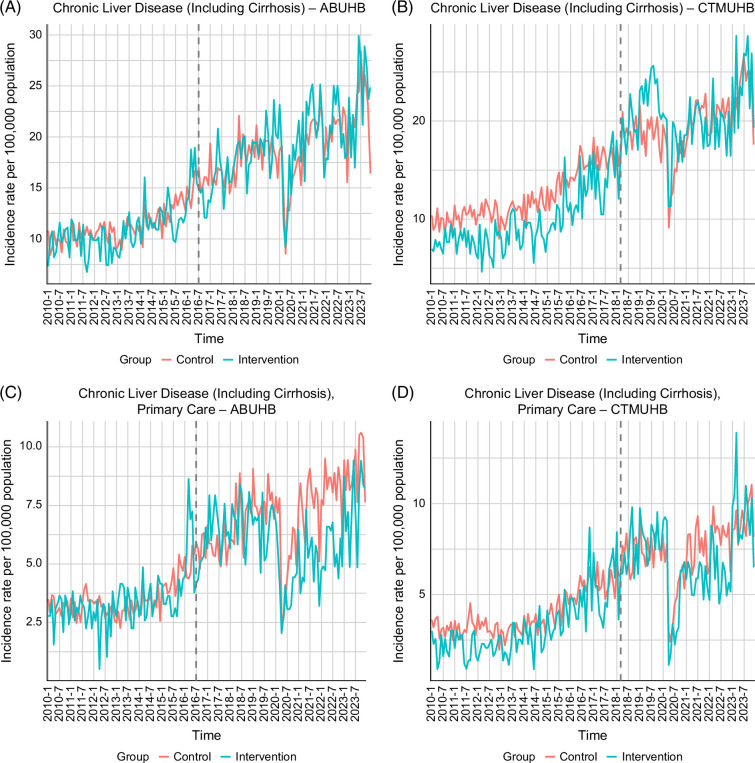
Incidence rate of chronic liver disease (including cirrhosis) in Aneurin Bevan University Health Board (ABUHB) and Cwm Taf Morgannwg University Health Board (CTMUHB) region. (A) Incidence rate of chronic liver disease (including cirrhosis) over time (ABUHB). (B) Incidence rate of chronic liver disease (including cirrhosis) over time (CTMUHB). (C) Incidence rate of chronic liver disease (including cirrhosis) diagnosed in primary care (ABUHB). (D) Incidence rate of chronic liver disease (including cirrhosis) diagnosed in primary care (CTMUHB).

In ABUHB, the average incidence of CLD (including cirrhosis) rose from 10.90 to 19.19 per 100,000 inhabitants (mean difference: 8.29, 95% CI: 6.34–10.24), while controls increased from 10.33 to 16.72 (mean difference: 6.39, 95% CI: 5.52–7.26) (Table 2). DiD analysis found no significant intervention effect on the detection of CLD (including cirrhosis) in ABUHB (IRR=1.09, 95% CI: 0.94–1.26, *p*=0.256; ATT=1.90, 95% CI: −1.44 to 5.24, *p*=0.204) (Table 3). In CTMUHB, the average incidence increased from 9.74 to 20.47 per 100,000 inhabitants (mean difference: 10.73, 95% CI: 8.75–12.70), compared to a rise from 11.15 to 17.40 in the control group (mean difference: 6.25, 95% CI: 5.37–7.14) (Table 2). DiD analysis indicated a significant intervention effect (IRR=1.35, 95% CI: 1.16–1.56, *p*<0.001; ATT=4.47, 95% CI: 1.25–7.69, *p*=0.016) (Table 3). When restricted to primary care only, no significant effect was detected in either health board (ABUHB: IRR=0.88, 95% CI: 0.75–1.03, *p*=0.111; ATT=−0.75, 95% CI: −2.87 to 1.37, *p*=0.405; CTMUHB: IRR=1.13, 95% CI: 0.99–1.29, *p*=0.066; ATT=0.36, 95% CI: −1.78 to 2.50, *p*=0.686) (Table 3). Trend-wise, ABUHB demonstrated no clear postimplementation divergence from controls. In CTMUHB, the intervention series was generally lower than the control series preimplementation; it then rose rapidly during the first 2 years after rollout, followed by attenuation after the COVID-19 lockdown. All panels showed a sharp drop around March 2020, aligning with the COVID-19 lockdown (Figure 3).

**TABLE 2:** Average incidence rate per 100,000 population before and after implementation in intervention and control groups for Aneurin Bevan and Cwm Taf Morgannwg University Health Boards

	Intervention, preimplementation^a^, mean (SD)	Intervention, postimplementation^b^, mean (SD)	Difference (95% CI)	Control, preimplementation, mean (SD)	Control, postimplementation, mean (SD)	Difference (95% CI)
Chronic liver disease (including cirrhosis)						
ABUHB	10.90 (2.38)	19.19 (4.02)	8.29 (6.34–10.24)	10.33 (5.50)	16.72 (7.84)	6.39 (5.52–7.26)
ABUHB, primary care	3.39 (1.17)	5.98 (1.56)	2.59 (1.82 – 3.36)	3.33 (1.66)	6.67 (3.37)	3.34 (3.00 – 3.69)
CTMUHB	9.74 (3.09)	20.47 (3.44)	10.73 (8.75–12.70)	11.15 (6.07)	17.40 (7.91)	6.25 (5.37–7.14)
CTMUHB, primary care	3.21 (1.63)	6.96 (2.24)	3.75 (2.95–4.54)	3.72 (2.02)	7.10 (3.47)	3.39 (3.03 – 3.75)
Cirrhosis only						
ABUHB	1.35 (0.50)	2.73 (0.72)	1.38 (1.06 – 1.70)	1.30 (0.92)	2.13 (1.28)	0.83 (0.68–0.97)
ABUHB, primary care	0.27 (0.23)	0.70 (0.29)	0.43 (0.32 – 0.53)	0.23 (0.28)	0.51 (0.42)	0.27 (0.23 – 0.32)
CTMUHB	1.79 (0.76)	3.21 (0.98)	1.43 (1.09 – 1.76)	1.42 (1.05)	2.20 (1.24)	0.78 (0.63–0.93)
CTMUHB, primary care	0.21 (0.20)	0.53 (0.40)	0.32 (0.21 – 0.43)	0.26 (0.33)	0.54 (0.42)	0.28 (0.23 – 0.33)

^a^ For ABUHB: preimplementation=January 1, 2010, to July 3, 2016; postimplementation=July 4, 2016, to December 31, 2023.

^b^ For CTMUHB: preimplementation=January 1, 2010, to February 28, 2018; postimplementation=March 1, 2018, to December 31, 2023.

Abbreviations: ABUHB, Aneurin Bevan University Health Board; CTMUHB, Cwm Taf Morgannwg University Health Board.

**TABLE 3:** Effect of introduction of reflex AST:ALT pathway for Aneurin Bevan and Cwm Taf Morgannwg University Health Boards

	Poisson regression model			Linear regression model			
	IRR	95% CI	*p*	ATT	95% CI	*p*	Parallel trends test
Chronic liver disease (including cirrhosis)							
ABUHB	1.09	0.94–1.26	0.256	1.90	−1.44–5.24	0.204	0.431
ABUHB, primary care	0.88	0.75–1.03	0.111	−0.75	−2.87–1.37	0.405	0.368
CTMUHB	1.35	1.16–1.56	<0.001	4.47	1.25–7.69	0.016	0.421
CTMUHB, primary care	1.13	0.99–1.29	0.066	0.36	−1.78–2.50	0.686	0.177
Cirrhosis only							
ABUHB	1.24	1.15–1.34	<0.001	0.56	0.06–1.06	0.036	0.521
ABUHB, primary care	1.18	1.02–1.37	0.024	0.16	−0.03–0.34	0.078	0.082
CTMUHB	1.16	1.02–1.33	0.028	0.65	0.24–1.06	0.010	0.691
CTMUHB, primary care	1.25	1.12–1.39	<0.001	0.04	−0.07–0.16	0.359	0.888

Abbreviations: ABUHB, Aneurin Bevan University Health Board; ATT, Average Treatment Effect on the Treated; CTMUHB, Cwm Taf Morgannwg University Health Board; IRR, incidence rate ratio.

### Compensated cirrhosis

**FIGURE 4:**
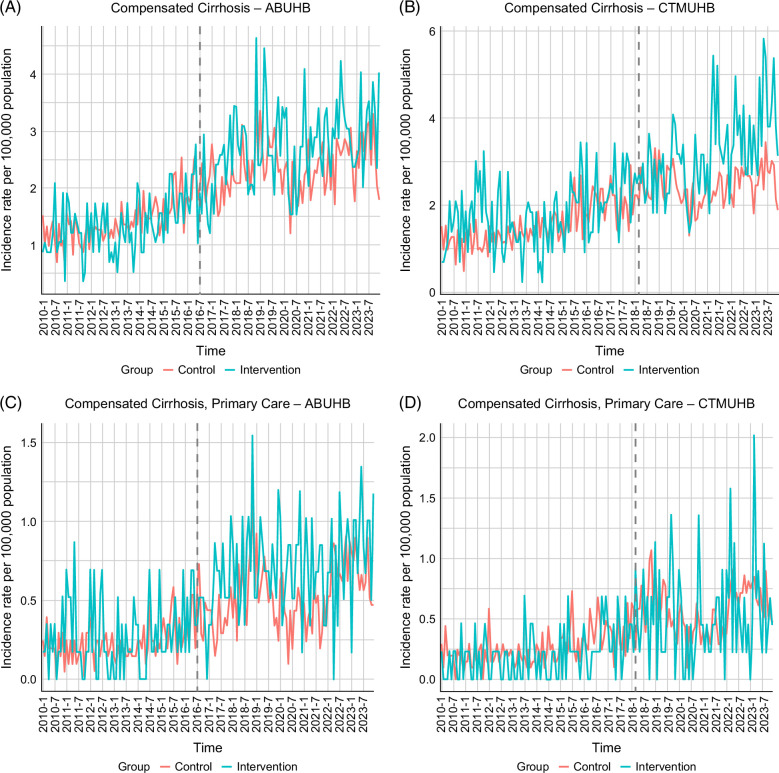
Incidence rate of cirrhosis in the ABUHB and CTMUHB region. (A) Incidence rate of cirrhosis over time (ABUHB). (B) Incidence rate of cirrhosis over time (CTMUHB). (C) Incidence rate of cirrhosis diagnosed in primary care (ABUHB); (D) Incidence rate of cirrhosis diagnosed in primary care (CTMUHB). Abbreviations: ABUHB, Aneurin Bevan University Health Board; CTMUHB, Cwm Taf Morgannwg University Health Board.

We conducted a subanalysis to evaluate changes in the incidence rate of compensated cirrhosis. In ABUHB, the average incidence rate increased from 1.35 to 2.73 per 100,000 inhabitants (mean difference: 1.38, 95% CI: 1.06–1.70), versus 1.30–2.13 in the control group (mean difference: 0.83, 95% CI: 0.68–0.97) (Table 2). DiD analysis indicated a significant intervention effect (IRR=1.24, 95% CI: 1.15–1.34, *p*<0.001; ATT=0.56, 95% CI: 0.06–1.06, *p*=0.036) (Table 3). In CTMUHB, incidence rose from 1.79 to 3.21 per 100,000 inhabitants (mean difference: 1.43, 95% CI: 1.09–1.76), compared with 1.42–2.20 in control groups (mean difference: 0.78, 95% CI: 0.63–0.93), with a similar significant DiD estimate for intervention effect (IRR=1.16, 95% CI: 1.02–1.33, *p*=0.028; ATT=0.65, 95% CI: 0.24–1.06, *p*=0.01) (Tables 2, 3). When restricted to primary care settings, only Poisson regression models detected a significant difference in both health boards (ABUHB: IRR=1.18, 95% CI: 1.02–1.37, *p*=0.024; ATT=0.16, 95% CI: −0.03 to 0.34, *p*=0.078; CTMUHB: IRR=1.25, 95% CI: 1.12–1.39, *p*<0.001; ATT=0.04, 95% CI: −0.07 to 0.16, *p*=0.359) (Table 3). Due to the low monthly count and high variance of cirrhosis diagnoses, the panels do not show a clear, sustained divergence between intervention and control. Nonetheless, after the implementation, the incidence rates in the intervention group exceed that of control in higher frequency and by a larger margin. A dip due to covid lockdown is visible around early 2020, though less marked than for CLD (including cirrhosis) diagnoses (Figure 4).

## DISCUSSION

Using a Difference-in-Difference approach, our study investigated the impact of introducing reflex AST testing and AAR calculation as a tool for improving early detection of liver disease in different regions in Wales. Our DiD analyses results showed a significant increase in cirrhosis incidence rate in both ABUHB and CTMUHB. We did not observe a significant change in the composite CLD (including cirrhosis) outcome in ABUHB. While CTMUHB demonstrated a significant increase in CLD (including cirrhosis) outcome, the rise occurred mainly within 2 years after implementation.

Over the past decade, numerous community-based pathways for the early detection of liver disease have been developed, many of which have demonstrated improvements in risk stratification and liver disease diagnosis.^14^ Our previous work, which introduced reflex AST testing and AAR calculation in ABUHB, observed an 81% increase in coded cirrhosis diagnoses within 2 years of implementation.^15^ Similarly, a community pathway using AAR ≥0.8 as a threshold for identifying at-risk patients suggested that 38.7% of liver disease cases would go undetected without an active case-finding strategy.^24^ In Germany, a structured screening initiative combining AAR and the AST-to-platelet ratio index was implemented and demonstrated a 59% higher likelihood of identifying early cirrhosis compared to routine care in patients without decompensated cirrhosis.^25^ Other implementations incorporating Fibrosis-4 as an initial screening tool have also demonstrated significant increases in liver disease detection rates.^26^^–^^28^ Additionally, a randomized trial using serum fibrosis markers and FibroScan reported more than double the number of liver disease diagnoses in the intervention group compared to controls.^29^ Beyond improved risk stratification and case detection, several studies have highlighted the cost-effectiveness and higher patient satisfaction associated with novel liver disease screening pathways.^30^^–^^32^ However, none of the existing studies has evaluated the long-term, population-level impact of such interventions. Most research has been limited to short follow-up periods (1–2 y postimplementation) and relatively small sample sizes, with the largest study including 17,770 participants as the screened population.^15^ In contrast, our study expands the investigation to a broader population, analyzing 78,917 individuals over a 14-year span of time before and after implementation.

As an extension of our previous work, we found significant increases in cirrhosis incidence in both ABUHB and CTMUHB when combining primary and secondary care. In primary care alone, effects were detected with Poisson but not with linear models. This may be due to the low case numbers captured at a monthly base in primary care. Because of the small numbers, we favor Poisson regression for this analysis as it is more appropriate for sparse count data. This finding is consistent with our observation of the trend visualization, where the intervention series exceeded the control more often and by a larger margin postintervention, despite the low counts and high variation. This result aligns with our earlier report of an 81% increase in cirrhosis in ABUHB in 2 years after implementation of AST:ALT pathway. Moreover, our study provided stronger evidence as it includes a control arm and evaluated changes in rates rather than raw counts, providing a more precise description of the changes in cirrhosis detection over the 14 years of follow-up.

We extended the outcome to the boarder range of CLD (including cirrhosis) to include underlying etiologies and fibrosis. CTMUHB demonstrated a significantly larger postimplementation change compared to controls, while ABUHB showed no significant difference. This heterogeneity is unlikely to be explained by demographic, socioeconomic, or geographic composition, as ABUHB and CTMUHB are neighboring health boards that shared similar age-sex distributions, which remained largely stable over follow-up. Nor is it readily explained by other policy changes, as the only liver disease relevant policy, the minimum unit pricing for alcohol, was implemented Wales-wide. One possible interpretation is variation in local implementation (eg, timing, laboratory workflows, referral practices), which may yield gains not only in cirrhosis detection but also in CLD coding in some settings. However, we cannot rule out the possibility that the difference was due to unmeasured confounding or random variation. The increase in CTMUHB was concentrated in the first 2 years postimplementation and attenuated after a COVID-19–related drop. We also observed that the incidence rate of CLD (including cirrhosis) of control groups occasionally exceeded intervention group in primary care settings. These features indicated an inconsistent, context-dependent effect on CLD (including cirrhosis) detection. Moreover, because the pathway primarily targets cirrhosis, any effect on CLD (including cirrhosis) detection remains speculative; the transient CLD (including cirrhosis) increase in CTMUHB should therefore be interpreted cautiously.

This study has several strengths. To our knowledge, this is the first study to evaluate the impact of a liver disease community pathway at the all-Wales population level over a long-term period, providing novel insights into its sustained effects. We employed a rigorous study design by incorporating DiD analysis along with intervention and comparator groups, allowing for a more robust evaluation of the intervention’s true effect. In addition, primary care and laboratory services are organized geographically at the health board level: patients register with local general practice and routine blood tests are processed by the same board’s hospital laboratory. This structure limits cross-board spillover of reflex AST testing and strengthens the internal validity of our comparisons. The use of large-scale national population data maximized the statistical power, improving the precision and reliability of our findings. Furthermore, this study leveraged a research-ready data asset constructed in the SAIL Databank and incorporated reproducible research pipelines. This enhanced the reproducibility and efficiency of our work and allowed future researchers to replicate findings or explore additional research questions using the same framework. However, some limitations should be acknowledged. Regulatory restrictions associated with data access agreements for data available within the SAIL Databank prevented the inclusion of sensitive conditions, such as HBV and HCV, which may have led to incomplete data for certain liver disease etiologies, potentially affecting the precision of our analysis. Additionally, the lack of data limited our ability to fully explore the reasons behind differences in intervention effects across health boards. Future research incorporating imaging, referral pathways and health care utilization patterns could provide a deeper understanding of regional variations in implementation and effectiveness.

## CONCLUSIONS

Our study provides the first long-term, all-Wales, population-level evaluation of a community liver disease pathway. Using a DiD approach, implementation of reflex AST:ALT testing was associated with significant increases in cirrhosis detection in both ABUHB and CTMUHB, and a short-term rise in the composite CLD (including cirrhosis) incidence in CTMUHB. This finding strengthens evidence that reflex AST:ALT testing can improve cirrhosis detection. Future research should examine each step of the clinical pathway—from test uptake and referral to imaging and diagnosis—to identify the key factors contributing to regional differences in intervention effectiveness and to inform strategies for optimizing and sustaining liver disease screening programs.

## Supplementary Material
