# Supplementary material for: A liver function test pathway significantly increases the early detection of chronic liver disease and cirrhosis
Source: Hepatol Commun. 2026 Jan 29;10(2):e0887. doi: 10.1097/HC9.0000000000000887 (PMC12858224; doi:10.1097/HC9.0000000000000887)
Supplement: Supplementary file 1 [file hc9-10-e0887-s001.docx]

**Supplemental Tables**

Supplemental Table 1 Code list for identifying liver disease phenotypes

| Phenotype | ICD-10 codes | Read codes |
| --- | --- | --- |
| Autoimmune liver disease | K754, K743, K831, K753 | J63B., J6141, J6160, J6617, J63X. |
| Haemochromatosis | E831 | C3500 |
| Metabolic liver disease | E880, E830 | C3762, C3761, C3510 |
| HBV ^*^ | B181, B180 |  |
| HCV ^*^ | B182 | A70E., A70F. |
| Alcohol-related liver disease | K70 | J613., J6130, J612., J6120, J610., J617., J6170, J611. |
| Non-alcoholic fatty liver disease | K760, K7581 | J61y1, J61y8 |
| Hepatitis not specified | K769, K7589, K73 | Jyu72, J614y |
| Congestive hepatopathy | K761, K762, K765 | J630., J636., J637. |
| Toxic liver disease | K71 | J635., J6350, J6351, J63252, J6353, J6354, J6355, J6356, J6357, J635X |
| Miscellaneous | K764, K768, K77 | J638., Jyu73, J63yz, Jyu75 |
| Hepatic fibrosis | K740, K741, K742 | J61y4, J61y6, J61y5 |
| Cirrhosis | K703, K744, K745, K746, K749 | J6161, J616z, J615z |
| Portal hypertension | K766, I81, I859, I982, I85 | J623., G81.., G8523, G852., G8521, G8522, G852z |
| *We identified Read codes(A7071, A7073, ZV02B, Q4091, 43B4., A7070, A7051, A7072, A70z0, A70A., A70B., A70C., A70D., A70E.,A70F., ZV02C) and ICD-10 codes (B180, B181, B182) for HBV and HCV. However, in order to comply with Data Protection Act 2018 and the UK General Data Protection Regulation, we could not include Read codes (A7071, A7073, ZV02B, Q4091, 43B4., A7070, A7051, A7072, A70z0, A70A., A70B., A70C., A70D., ZV02C) and ICD-10 codes (B171) as these were flagged as sensitive in the latest version of known sensitive code list of SAIL Databank  Abbreviations: HCV, hepatitis C virus; HBV, hepatitis B virus; ICD-10, International Classification of Diseases, 10th Revision; SAIL, Secure Anonymised Information Linkage. | | |
